# The Time Course of Segmentation and Cue-Selectivity in the Human Visual Cortex

**DOI:** 10.1371/journal.pone.0034205

**Published:** 2012-03-27

**Authors:** Lawrence G. Appelbaum, Justin M. Ales, Anthony M. Norcia

**Affiliations:** 1 Center for Cognitive Neuroscience, Duke University, Durham, North Carolina, United States of America; 2 Department of Psychiatry, Duke University, Durham, North Carolina, United States of America; 3 Department of Psychology, Stanford University, Stanford, California, United States of America; University Of Cambridge, United Kingdom

## Abstract

Texture discontinuities are a fundamental cue by which the visual system segments objects from their background. The neural mechanisms supporting texture-based segmentation are therefore critical to visual perception and cognition. In the present experiment we employ an EEG source-imaging approach in order to study the time course of texture-based segmentation in the human brain. Visual Evoked Potentials were recorded to four types of stimuli in which periodic temporal modulation of a central 3° figure region could either support figure-ground segmentation, or have identical local texture modulations but not produce changes in global image segmentation. The image discontinuities were defined either by orientation or phase differences across image regions. Evoked responses to these four stimuli were analyzed both at the scalp and on the cortical surface in retinotopic and functional regions-of-interest (ROIs) defined separately using fMRI on a subject-by-subject basis. Texture segmentation (tsVEP: segmenting versus non-segmenting) and cue-specific (csVEP: orientation versus phase) responses exhibited distinctive patterns of activity. Alternations between uniform and segmented images produced highly asymmetric responses that were larger after transitions from the uniform to the segmented state. Texture modulations that signaled the appearance of a figure evoked a pattern of increased activity starting at ∼143 ms that was larger in V1 and LOC ROIs, relative to identical modulations that didn't signal figure-ground segmentation. This segmentation-related activity occurred after an initial response phase that did not depend on the global segmentation structure of the image. The two cue types evoked similar tsVEPs up to 230 ms when they differed in the V4 and LOC ROIs. The evolution of the response proceeded largely in the feed-forward direction, with only weak evidence for feedback-related activity.

## Introduction

The boundaries between objects and their supporting backgrounds, or between surfaces at different depths, create discontinuities in feature maps of orientation, relative alignment, motion, disparity, color and spatial scale. The detection of these region boundaries, defined by differences in local texture features and the integration of surface information within these boundaries, provide an initial pre-attentive parsing of the visual scene [1]–[5].

The early stages of the scene segmentation process have been extensively studied in humans and primates using textured stimuli into which discontinuities in single visual features have been embedded. A common finding in the single-unit literature has been that the response to an isolated feature presented within the classical receptive field is suppressed by the addition of texture in the cell's non-classical surround [6], [7], [8], [9]. The magnitude of the suppression is generally largest for homogeneous textures, such as those whose elements are all of the same orientation [10]–[18]. Because of this, surround suppression or “end-stopping” has been proposed as a mechanism by which cells in the early visual pathway could signal feature discontinuity [18]–[20].

A number of single unit studies conducted in primary visual cortex have compared the magnitude of surround suppression for iso-oriented center and surround configurations comprised of stimuli that are either continuous or mis-aligned (discontinuous). Some of these studies have found that surround suppression is relatively unaffected [11], [17], [18] by the relative alignment of center and surround, but others have found significant effects [21]–[23] or a mixture of effects in different cells, with some cell showing more suppression for aligned stimuli and other misaligned ones [14]. The largest modulatory effects of surround alignment thus far reported have been found in alert macaque [23] where sensitivity to the relative phase of center and surround was also correlated with an independent measure of the strength of surround suppression [23].

Human observers are extremely sensitive to alignment discontinuities in oriented textures – thresholds for vernier offset discontinuities are as low as a few arc seconds and are among the finest discriminations made by the visual system [24]. The existing literature on V1 sensitivity to relative alignment reviewed above is quite mixed and the contribution is of early visual cortex to the processing of this cue is at present unclear. It is also unclear how strongly represented the cue of relative alignment is compared to other cues for segmentation such as the orientation difference cue which has proven to be robust across studies in V1. Moreover, the image segmentation process has only been studied in detail in V1 using single-unit recording, but fMRI studies in humans indicate wide-spread activity is present in extra-striate cortical areas [1], [25], [26]

Previous studies from our laboratory have used a frequency tagging technique and ROI-based EEG source-imaging approach to study texture segmentation [27]–[29]. In these studies a large background texture was modulated at 3.6 Hz and a smaller “figure” region was modulated at 3.0 Hz. These studies used both orientation and alignment texture cues and found that the evoked responses attributed to the figure regions were substantially similar, independent of the defining cue, and proceeded to a large extent independent of attentional allocation [30]. The two-frequency method was useful for defining separate region responses and region interaction responses, but it was not possible to define the sequential order of processing relative to the onset of the segmentation cue, as had been done in previous tsVEP studies.

In the present study we exploited a refined version of this ROI-based EEG source-imaging approach [31], [32] with stimuli that modulated at a much slower rate in order to examine the time course and source distribution of the tsVEP generated by orientation and alignment cues. This approach allowed us to assess how texture-based segmentation proceeds through both retinotopic visual areas, as well as, areas of lateral cortex previously implicated in object processing [33], [34], and to evaluate differences in these responses due to the defining texture cues. The results are presented first with regard to the visual evoked potentials (VEPs) as they are quantified at the scalp, and then on the cortical surface in retinotopic and functional regions-of-interest (ROIs) defined separately using fMRI on a subject-by-subject basis. This study therefore provides the first quantitative measurements of the relative strength and precise timing of segmentation-related activity as it is propagated throughout the human visual cortical hierarchy.

## Materials and Methods

### Ethics Statement

Written informed consent was obtained prior to the study under a protocol that was approved by the Institutional Review Board of the California Pacific Medical Center.

### Participants

Fifteen neurologically typical individuals (mean age 34, 13 male) with normal, or corrected-to-normal, visual acuity participated in this experiment.

### Visual Stimuli and Task

Stimuli in the present experiment were designed such that periodic temporal modulation of a central ‘figure’ region could either support figure-ground segmentation, or have identical local texture modulations but not produce changes in global image segmentation. In these stimuli, the central 3° of a one-dimensional, random-luminance texture (minimum bar width 2 arc min, maximum contrast 90%) modulated at 1 Hz while the remaining 12.6°×12.6° horizontal ‘background’ texture field stayed stationary. The texture within the central disk modulated either in its relative orientation or phase with respect to the background, thus creating a 2×2 experimental design defined by the segmentation state (changing versus constant) and the cue type (orientation versus phase).

**Figure 1:**
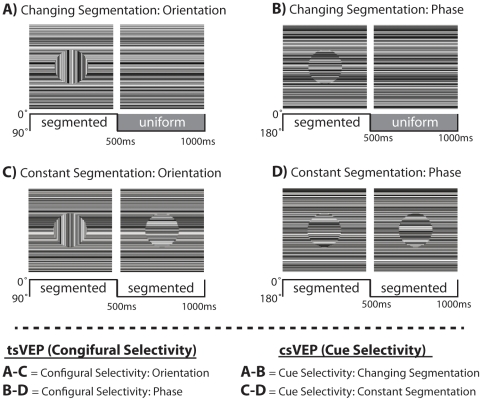
Schematic representation of the four stimulus conditions. Two frames of each stimulus are shown above a schematic of that stimulus' segmentation sequence. A) Orientation-defined forms and B) phase-defined forms either alternated segmentation states every 500 ms (top) or remained constantly segmented throughout the trial (C and D). The experimental contrasts defining configural and cue selectivity are shown below.

Two frames from each stimulus type are shown in **Figure 1A–D**. In panels A and C, an ‘*orientation-defined form*’ is shown in which the texture in the central disk changed orientation by 90° every 500 ms. In panels B and D a ‘*phase-defined form*’ is shown in which the texture in the central disk alternated phase (180° rotation, flipping about the midline) every 500 ms. For each of these two cue types, we presented stimuli that either alternated their segmentation state, or else remained constantly segmented. Panels A and B show examples of stimuli that alternate between a uniform field and a segmented one. Because the global figure-ground configuration of these stimuli changed every 500 ms, we refer to these conditions as ‘*changing segmentation*’ conditions. To evaluate brain mechanisms selective to the global segmentation state of the display (see “Quantitative and statistical analyses” section below), control stimuli with identical local pattern changes, but no changes in global segmentation were also presented for each cue type. In these stimuli, shown in panels C and D, the figure and background regions were composed of different random luminance textures and therefore the figure never blended into the background, but the local temporal transients and the temporal discontinuity between regions was still present. We refer to these stimuli as ‘*constantly segmented*’ as a border was always present between regions, and thus there was never a uniform state. Below each stimulus illustration in **Figure 1** is a schematic representation of the temporal sequence of the figure-background segmentation. In these representations, the orientations of the figure region textures (0° vs. 90°; 0° vs. 180°) are depicted by the solid lines while the sequence of segmentation states are indicated by the shaded gray (“uniform”) and white (“segmented”) areas.

In order to control for possible response variability contributed by changes in attentional state or strategy during the recording, 12 of the 15 participants were instructed to fixate a mark at the center of the display and to indicate occasional changes in the contrast of the textured display with a button press. On 20% of the one-second stimulus cycles, the contrast of the entire display was reduced by 10%. The remaining 3 subjects were instructed to maintain central fixation and distribute attention evenly over the entire display. No behavioral responses were collected for these participants and their electrophysiological data did not differ qualitatively from the remaining participants.

Stimuli were generated on a Power Macintosh G4 running the in-house PowerDiva software suite and were presented on a Westinghouse model LTV32w1 LCD video monitor at a resolution of 1360×768 pixels, and a 59.8 Hz vertical refresh rate. The full display subtended 16.6° by 12.6° with an average luminance of 119 cd/m^2^. Stimuli were viewed binocularly in a dark and quiet room as whole head, 128-channel EEG was simultaneously recorded. Individual trials lasted 16 seconds and conditions were randomized within a block. A typical session lasted roughly 45 minutes and consisted of 1 or 2 practice blocks followed by 20 blocks of randomized trials in which the observer paced the presentation and was given opportunity to rest between blocks.

### EEG signal acquisition

The procedures for EEG signal acquisition, head conductivity modeling, and visual area definition are similar to those utilized in our previous studies and are described in brief here [28], [29], [31], [35]. As in our previous experiments, the EEG was collected with 128-sensor HydroCell Sensor Nets (Electrical Geodesics, Eugene OR), 0.1 Hz high-pass and 50.0 Hz (Bessel) low-pass filtered, and digitized at 432 Hz with a precision of 4-bits per microvolt at the input. The 16-bit analog-to-digital converter was externally clocked via a hardware interface to the video card that used the horizontal synch of the video monitor as its base clock. Following each experimental session, the 3D locations of all electrodes and three major fiducials (nasion, left and right peri-auricular points) were digitized using a 3Space Fastrack 3-D digitizer (Polhemus, Colchester, VT). For all observers, the 3D digitized locations were used to co-register the electrodes to their T1-weighted anatomical MRI scans.

Raw data were evaluated off-line according to a sample-by-sample thresholding procedure to remove noisy sensors that were replaced by the average of the six nearest spatial neighbors. Once noisy sensors were substituted, the EEG was re-referenced to the common average of all the sensors. Additionally, EEG epochs that contained a large percentage of data samples exceeding threshold (25–50 micro volts) were excluded on a sensor-by-sensor basis (<15% of epochs). Time-averages for each stimulus condition were computed over one stimulus cycle of 1.00 second.

### Source-imaging data acquisition and processing

#### Structural and Functional Magnetic Resonance Imaging (MRI)

Structural and functional MRI scanning was conducted at 3T (Siemens Tim Trio, Erlangen, Germany) using a 12-channel head coil. We acquired a T1-weighted MRI dataset (3-D MP-RAGE sequence, 0.8×0.8×0.8 mm3 and a 3-D T2-weighted dataset (SE sequence at 1×1×1 mm3 resolution) for tissue segmentation and registration with the functional scans. For fMRI, we employed a single-shot, gradient-echo EPI sequence (TR/TE = 2000/28 ms, flip angle 80, 126 volumes per run) with a voxel size of 1.7×1.7×2 mm3 (128×128 acquisition matrix, 220 mm FOV, bandwidth 1860 Hz/pixel, echo spacing 0.71 ms). We acquired 30 slices without gaps, positioned in the transverse-to-coronal plane approximately parallel to the corpus callosum and covering the whole cerebrum. Once per session, a 2-D SE T1-weighted volume was acquired with the same slice specifications as the functional series in order to facilitate registration of the fMRI data to the anatomical scan.

The FreeSurfer software package (http://surfer.nmr.mgh.harvard.edu) was used to perform gray and white matter segmentation and a mid-gray cortical surface extraction. This cortical surface had 20,484 isotropically spaced vertices and was used both as a source constraint and for defining the visual areas. The FreeSurfer package extracts both gray/white and gray/cerebrospinal fluid (CSF) boundaries, but these surfaces can have different surface orientations. In particular, the gray/white boundary has sharp gyri (the curvature changes rapidly) and smooth sulci (slowly changing surface curvature), while the gray/CSF boundary is the inverse, with smooth gyri and sharp sulci. In order to avoid these discontinuities, we generated a surface partway between these two boundaries that has gyri and sulci with approximately equal curvature.

Individual Boundary Element Method (BEM) conductivity models were derived from the T1 and T2 weighted MRI scans of each observer. The FSL toolbox (http://www.fmrib.ox.ac.uk/fsl/) was also used to segment contiguous volume regions for the scalp, outer skull, and inner skull and to convert these MRI volumes into inner skull, outer skull, and scalp surfaces [36], [37].

#### Visual area definition

Rotating wedge stimuli were used to map polar angle sensitivity and expanding and contracting ring stimuli were used to map retinal eccentricity up to 3.5°. Complete cycles lasted 24 sec and a total of 10 cycles in each of 3 scans were collected in each participant. Fourier analysis was used to extract the magnitude and phase of the BOLD signal, which was visualized on a flattened representation of the cortical surface. Retinotopic field mapping produced regions-of-interest (ROIs) defined for each participant's visual cortical areas V1, V2v, V2d, V3v, V3d in each hemisphere [38]. ROIs corresponding to each participant's human middle temporal area (hMT+) were identified, using low-contrast motion stimuli similar to those described by Huk et al. (2002). A contrast between scrambled versus intact objects (block design 12 sec intact/12 sec scrambled; 10 cycles (240 sec per scan, 2 to 3 scans) was used to define the LOC. The stimuli of [39] were used. These stimuli result in an activation that extends onto both the lateral and ventral surfaces [40]. Only the portion lying on the lateral surface, posterior and adjacent to hMT+ was included in our definition. Activations in ventral areas were more variable and sources in these areas are less visible in the EEG due to their greater depth.

#### Cortically constrained inverse

An L2 minimum norm inverse was computed with sources constrained to the location and orientation of the cortical surface. In addition, we modified the source covariance matrix in two ways to decrease the tendency of the minimum norm procedure to place sources outside of the visual areas. These constraints involved; 1) increasing the variance allowed within the visual areas by a factor of two relative to other vertices, and 2) enforcement of a local smoothness constraint within an area using the first- and second-order neighborhoods on the mesh with a weighting function equal to 0.5 for the first-order and 0.25 for the second-order relationships [31]. The smoothness constraint therefore respects areal boundaries unlike other smoothing methods such as LORETA that apply the same smoothing rule throughout cortex. Given this cortically constrained activity we estimated the response magnitude from each ROI by coherently averaging across all source locations within that ROI.

### Quantitative and statistical analyses

This experimental design consists of orientation- and phase-modulating stimuli that do (**Figure 1A and 1B**), or do not (**Figure 1C and 1D**), define a segmented circular figure. By contrasting these four stimuli we are able to isolate distinct brain responses that reflect neural selectivity for the segmentation state, as well as the defining texture cues of the scene. Specifically we consider two types of VEP differences (see bottom panel of **Figure 1**); the texture segmentation visually evoked potential (tsVEP) and the cue-specific visually evoked potential (csVEP). To arrive at the tsVEP, responses of the constantly segmented stimuli were subtracted from the changing segmentation stimuli for each cue type. Thus to arrive at the tsVEP for orientation, responses to the Constant Segmentation: Orientation stimuli (Figure 1C) were subtracted from the Changing Segmentation: Orientation (Figure 1A) stimuli, and likewise for B–D. In this subtraction, the responses to the low-level features relating to the stimulus transients (texture orientation or phase changes) that are common to the two conditions subtract out, isolating aspects of processing that are specific to the appearance and disappearance of the segmented form, or alternatively the importance of continuous, collinear texture [41], [42]. To arrive at the csVEP, responses for the phase-defined form stimuli were subtracted from the orientation-defined form stimuli, separately for the changing (A–B) and constant (C–D) segmentation conditions. These subtractions isolate responses selective for the defining texture cues.

Quantitative and statistical analyses were carried out in sensor space and source space by means of a 2×2 comparison between the configural- and cue-selectivity. For this purpose we computed the global field power (GFP) over 1 second (1 cycle) of each stimulus type. The GFP is a measure of the spatial variation of the potential measured at each point in time over all 128 channels (**Eq 1**) and represents the spatial standard deviation of the whole map activity. Since ERP topographies tend to remain stable for short periods of time, and typically change at points with relatively low GFP [43] this measure provides an intuitive means for assessing the spatio-temporal sequence of activity elicited by our stimuli. In the following equation for the GFP V_i_(t) is the voltage measured at time t for the single electrode i, N is the total number of electrodes.
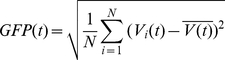
(1)


In order to focus the statistical questions on differences caused by the shape of the temporal waveforms, and discount each subjects' global amplitude scaling, we used z-score normalization. For each subject we calculated the standard deviation over all time points within an analysis group (either GFP, or source space ROI). This standard deviation (two estimates per subject, one for GFP, one for source space) was used to divide all waveforms with an analysis group and provide a normalized response measure.

Differences between the experimental conditions were identified by t-tests, and a permutation test based on methods devised by Blair and Karniski [44] and described in detail in Appelbaum et al. [29]. Briefly, the null hypothesis is that there is no difference between conditions. To test this hypothesis we make synthetic datasets in which the condition labels for an individual subject's data have been randomly permuted. For each permutation we calculate t-scores of the difference and find the longest run of consecutive time points with p-values less than .05. This procedure provides a nonparametric reference distribution of consecutive significant p-values. We rejected the null hypothesis if the length of any consecutive sequence of significant *t*-scores in the original, nonrandomized data exceeded *95%* of the values in the null distribution. Because each permutation sample contributes only its *longest* significant sequence to the reference distribution this procedure implicitly compensates for the problem of multiple comparisons, and is a valid test for the omnibus hypothesis of no difference between the waveforms at any time point. Furthermore, this test not only detects significant departures from the null hypothesis, it also localizes the time periods when such departures occur. However, since the correction procedure is tied to the length of the data and the somewhat arbitrary choice of keeping family-wise error at 5%, we therefore also present the uncorrected significance values (see red/yellow color maps displaying “uncorrected p-Values”). By evaluating the data using both statistical approaches, we are better able to identify time periods when the responses depart from the null hypothesis.

## Results

### Behavioral results

To ensure that attention was deployed consistently during the viewing of the stimuli, subjects were instructed to detect subtle changes in the contrast of the stimulus texture that occurred randomly on 20% of the trials. In this task, subjects demonstrated a high level of accuracy, correctly identifying 94% of the contrast decrement targets. It can therefore be inferred that subjects were consistently vigilant and attentive to the presentation of the segmenting stimuli.

### Sensor-Space Visual Evoked Potential results

In the present experiment we compare the visually evoked potentials to four types of 1 Hz, texture-modulating stimuli. Our description of these results proceeds first with a ‘sensor-space’ analysis (voltage as a function of electrode location) by describing the waveform morphology and global-field power (GFP) for the orientation-defined form and then each of the configuration and cue contrasts, as well as their double difference. We then show source analyses of waveforms derived from individually defined visual area ROI's (V1, V3A, V4, MT, and LOC) that are each sufficiently separated to be resolvable by our inverse method [31].

### Waveform Morphology and Global Field Power

**Figure 2:**
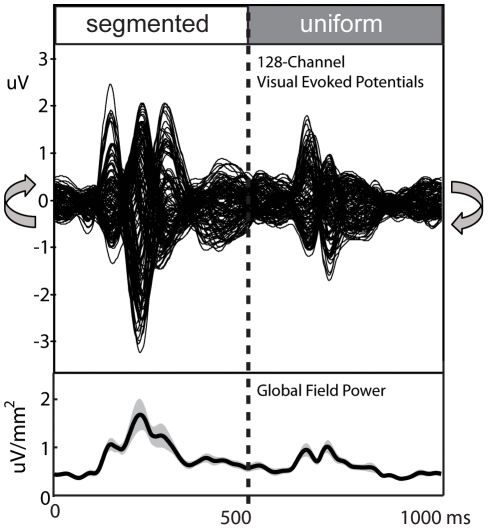
Grand average waveforms and global field power for the orientation-defined form response. Prominent responses are present to the both the appearance (left) and disappearance (right) of the orientation-defined figure. Responses are larger and more protracted following the onset of the figure region than the return to a uniform state. Curved arrows to the left and right of the waveforms indicate that this is the response to a periodic stimulus. The grey shading around the Global Field Power trace indicates +/−1 standard error of measurement for the mean GFP.

**Figure 3:**
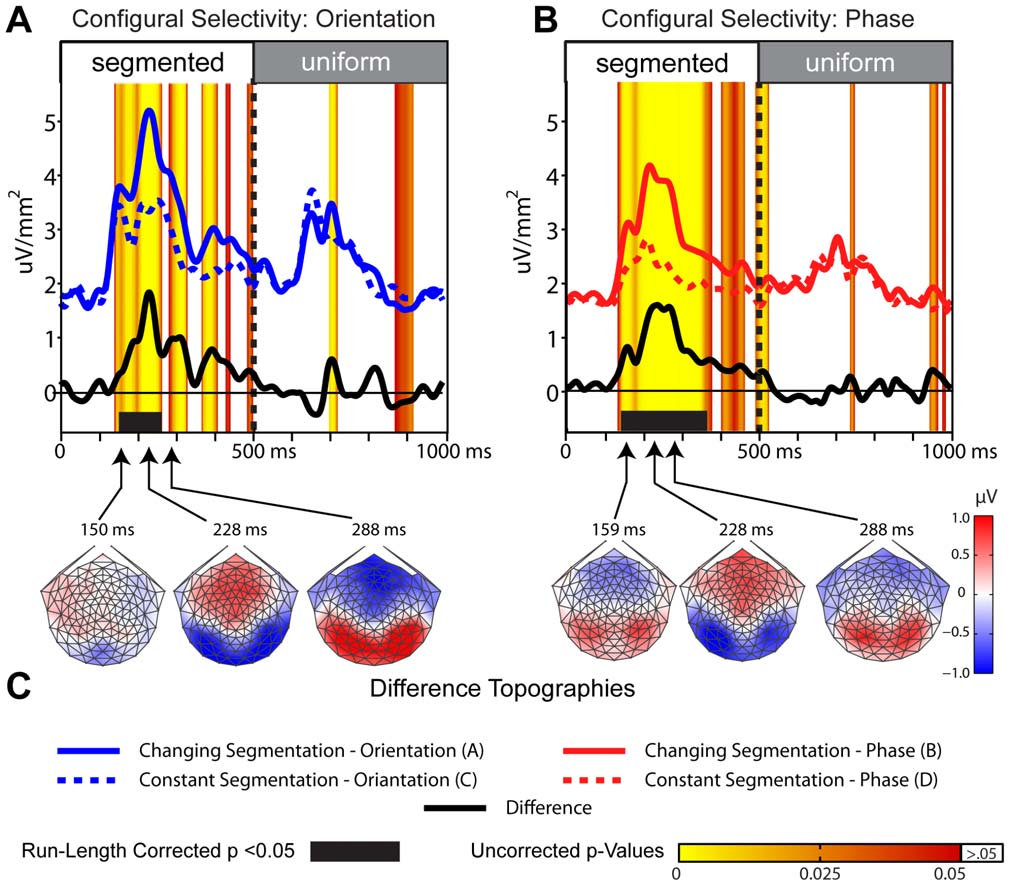
Sensor-space configural selectivity contrasts. Global field power, GFP differences, and corrected and uncorrected significance levels for the (**A**) orientation-defined and (**B**) phase-defined configuration contrasts. Changing segmentation (solid), constant segmentation (dashed), and differences (black) waveforms are shown for the orientation- (blue) and phase-defined (red) stimuli. Intervals reaching significance (p = .05) according to run-length corrected permutation tests are indicated by the black bars. Uncorrected significance values are depicted by the red-yellow color scale, starting at p<.05-level. Values higher than the .05-level are plotted as white. (**C**) Shows the topographic distributions of the difference waves at the three peaks in GFP after figure onset for each of the two configuration contrasts.

For all subjects, prominent responses were present after each of the two texture transients in the stimulus cycle. Grand average evoked responses are shown for the orientation-defined form, changing segmentation condition in **Figure 2**. Here one cycle (1 second) of the grand average waveforms for all 128-channels are shown superimposed on top of each other and are presented above the global field power (GFP) of the response. As seen in both the waveforms and the GFP, the appearance and disappearance of a segmented figure results in a highly asymmetric response. Responses to the appearance of the segmented figure in the first half of the stimulus cycle (0–500 ms) are larger and more protracted than those to the disappearance of the figure, in the second half of the cycle (500–1000 ms). In particular, the appearance produces three high-amplitude peaks of activity, while the disappearance produces responses of lesser signal amplitude with only two peaks. In both cases, the evoked responses produce bilateral, occipital distributions that differ in polarity at successive peaks (see **Figure 3**).

### Sensor-space differences due to configuration

For both cue types, cortical responses evoked by the changing and constant segmentation stimuli differed after the image updates at 0 ms, but not after those at 500 ms (e.g. the times of appearance and disappearance of the figure in the changing segmentation condition, respectively). As shown in **Figure 3A** for the orientation-defined textures and **Figure 3B** for the phase-defined textures, the changing-segmentation stimuli evoked responses of greater amplitude than responses to the corresponding constant segmentation stimuli for protracted periods following the onset of the segmented figure at 0 ms (solid and dashed curves, respectively). Configural selectivity, as determined by run-length corrected permutation tests comparing the changing versus the constant segmentation stimuli, was present from 148–262 ms for the orientation-defined textures (panel A) and from 143–365 ms for the phase-defined (panel B) textures (see black bars). For reference the uncorrected, sample-by-sample p-values are depicted by the yellow-red color map overlays for both configuration comparisons.

The two cue types produced qualitatively different topographic distributions after figure onset as shown in **Figure 3C** by the three topographic distributions plotted below the GFP traces. Whereas the onset of a phase-defined figure produced an initial bilateral component that peaked at 159 ms, the orientation-defined figure produced a lower amplitude difference that was more medial occipital in its initial focus. Scalp topographies at later time points (228 and 288 ms) did not differ substantially as a function of the defining cue type. These difference-wave distributions are explored further below in an ROI-based source space analysis, below.

### Sensor-space differences due to cue type

**Figure 4:**
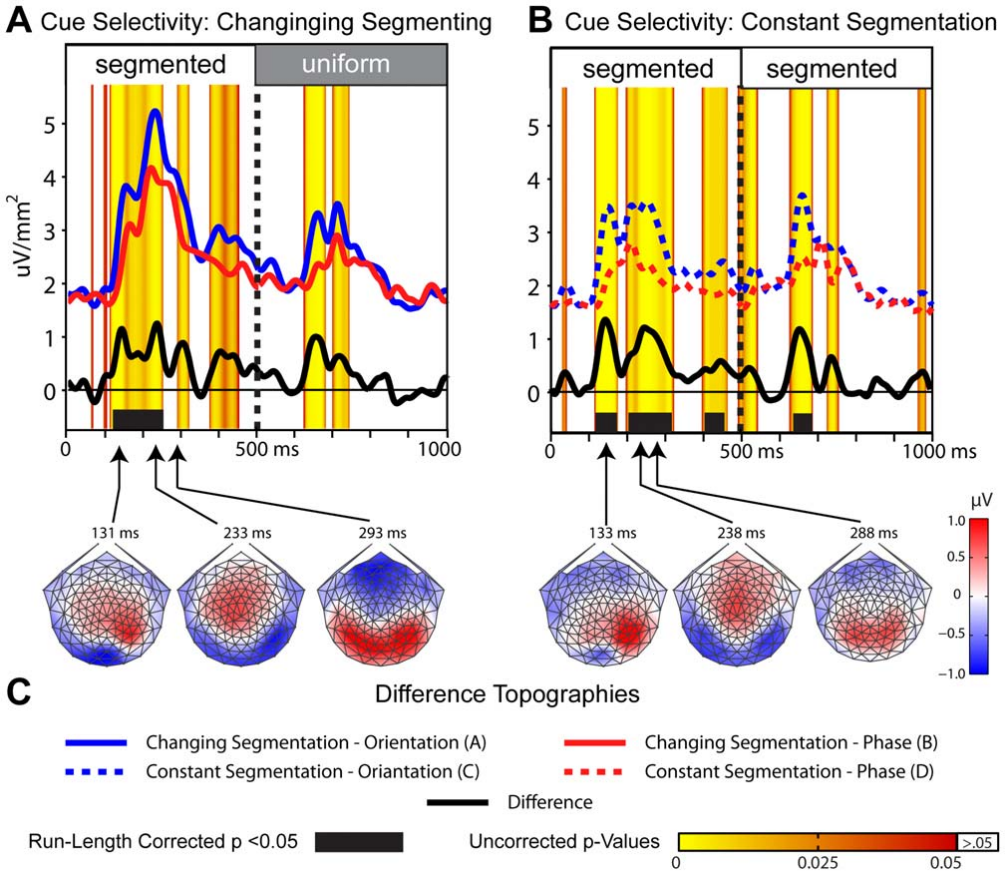
Sensor-space cue selectivity contrasts. Global field power, GFP-differences, and corrected and uncorrected significance levels for differences in the defining texture cue when these cues (**A**) changed segmentation states, or (**B**) remained constantly segmented. (**C**) Shows the topographic distributions of the difference waves at the three peaks in GFP after the texture transient for each of the two cue contrasts.

Cortical responses evoked by the changing and constantly segmenting stimuli also differed as a function of the defining cue type. As shown in **Figure 4A and 4B**, the GFP evoked by the orientation-defined stimuli (blue) produced greater amplitude responses that those evoked by the phase-defined stimuli (red) both when these textures supported segmentation, and not. Statistically significant differences were present between the two changing segmenting textures from 119–253 ms following the appearance of the figure. Significant differences in cue selectivity were present to the constantly segmented stimuli from 119–174; 205–322; and 406–463 ms during the segmented phase of the cycle, and also from 636–689 ms of the uniform phase of the response (i.e. 136–189 ms following the return to the uniform state). In general, texture-cue differences were present during both segmented and uniform phases of the response cycle as indicated by cluster of high, uncorrected significance levels between 600 and 750 ms.

### Source-Space Visual Evoked Potential results

**Figure 5:**
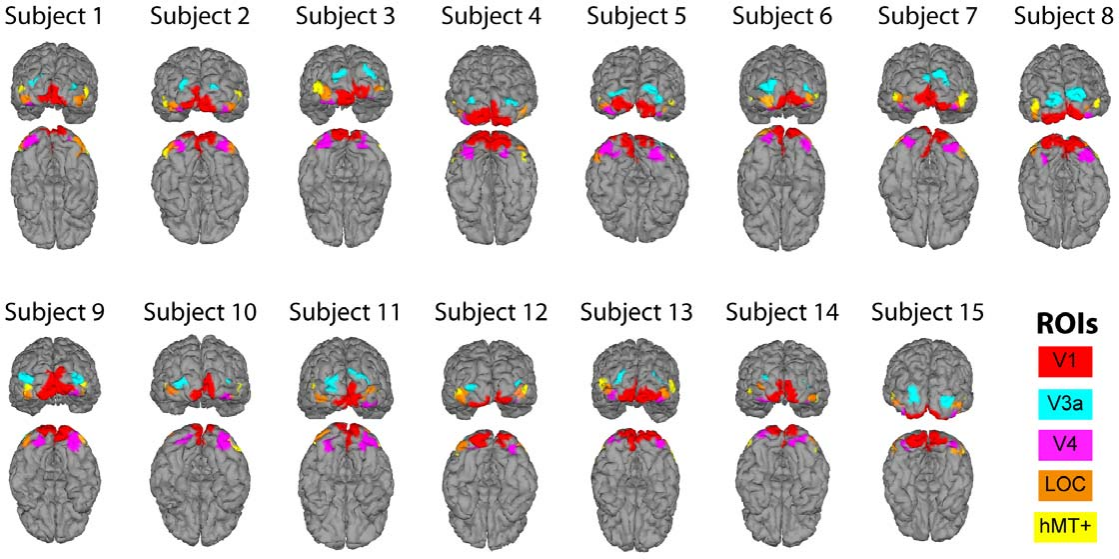
ROI locations for all subjects color coded and shown from posterior and inferior perspectives.

In order to quantitatively assess the differences in configuration- and cue-selectivity in the brain, we performed a region-of-interest (ROI) analysis on the time-averaged responses, focusing on the first half of the response cycle where selectivity was greatest in the sensor-space results (i.e., 0–500 ms). Source current density reconstructions were computed for five ROIs centered on well-separated visual areas. These regions; LOC, hMT+, V4, V3a, and V1 are depicted for each individual subject in **Figure 5**, are located on the lateral surface, the dorsal surface, the ventral surface, and the posterior pole, and can be defined reliably in all subjects.

### Source-space differences due to configuration

The appearance of both orientation- and phase-defined stimuli evoked differential activity for the changing versus constant segmentation stimuli across many of the measured ROIs. These patterns of selectivity for the two cue-types both shared early run-length corrected activity in V1 and later selectivity in the LOC ROI. The dynamics of configural selectivity however, differed substantially across other ROIs and latencies (see **Table 1** for specific latencies).

**Table 1:** Start and stop times for the run-length corrected permutation tests (depicted by the black bars in Figures 6 & 7).

	*Configural Selectivity Orientation*	*Configural Selectivity Phase*	*Cue Selectivity Changing Segmentation*	*Cue Selectivity Constant Segmentation*
***LOC***	*255–339*	*281–332*	*250–334*	*186–248*
***MT***	*none*	*none*	*none*	*none*
***V4***	*212–248*	*none*	*none*	*none*
***V3A***	*none*	*291–355*	*241–296*	*none*
***V1***	*133–181*	*143–183*	*124–169*	*114–250*

**Figure 6:**
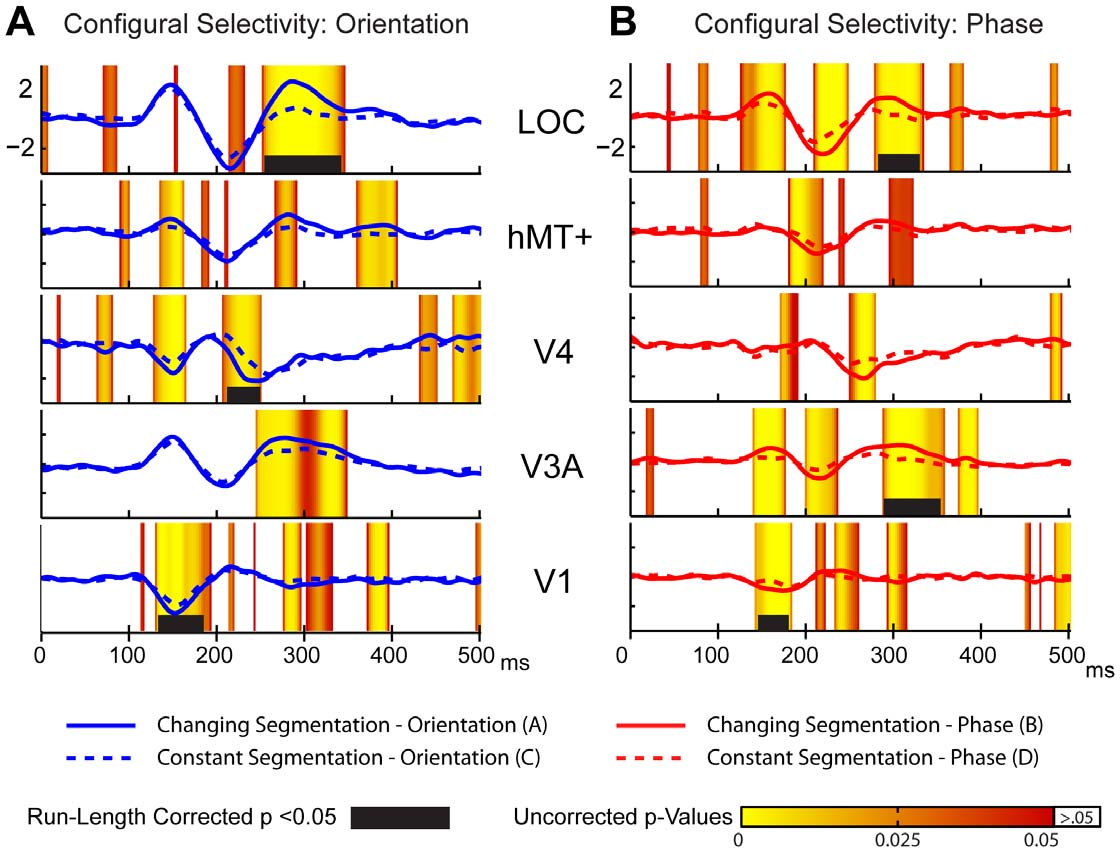
Source-space configural selectivity contrasts. Responses waveforms in 5 visual cortical areas showing configural selectivity to the appearance (2^nd^ half cycle) of the (**A**) orientation and (**B**) phase defined forms. Global field power for the changing (solid) and constant (dashed) configurations of the orientation-defined (blue) and phase-defined (red) stimuli.

For the orientation-defined stimuli (**Figure 6A**), run-corrected differences emerged in V1 from 133–183 ms after figure onset. Orientation-defined stimuli also produced selective activity that didn't reach run-length-corrected significance levels in the V4 and MT ROIs over this latency range. Early configural selectivity for the phase-defined stimuli (**Figure 6b**) was present from 143–183 ms, according to run-length corrected criteria in V1, but also at uncorrected levels in the V3A and LOC ROIs at these early latency ranges. For both cues, this early configuration selectivity occurs on the rising phase of the activity evoked by the segmenting texture transient. As seen in the first difference topography for each contrast in **Figure 3c** (150 and 159 ms), this difference is more lateral and negative in polarity for the phase-defined contrast, and more central and positive for the orientation-defined segmentation, and is in agreement with the underlying pattern of ROI activations.

At the time of the second GFP peak (228 ms), the scalp topography for both cue types comprises bilateral negative occipital potentials. Over this time range differential activity is present at the corrected criterion in the V4 ROI (212 to 248 ms) for the orientation-defined stimuli. In contrast, at the same latency no ROIs reached run-length corrected criteria for the phase-defined stimuli, but moderate levels of uncorrected selectivity were present in the V3A and hMT+ ROIs.

Later activity depicted by the 3^rd^ topographic map for each contrast produced run-length corrected selectivity in the LOC ROI from 255–339 ms and 281–332 for the orientation and phase stimuli, respectively. Configural selectivity was also present in the V3A ROI over this same period, reached corrected significance levels for the phase stimuli (291–355 ms), and was robust but not significant for the orientation-defined stimuli. In addition, there were sporadic periods of differential activity in the V1 ROI over this latency range that did not meet corrected criteria. Overall, the phase-defined configuration exhibited more periods of differential segmentation-related activity than did the orientation-defined configuration, including activity in the V3A and hMT+ ROIs that was not present with the orientation-defined stimuli.

### Source-space differences due to cue type

**Figure 7:**
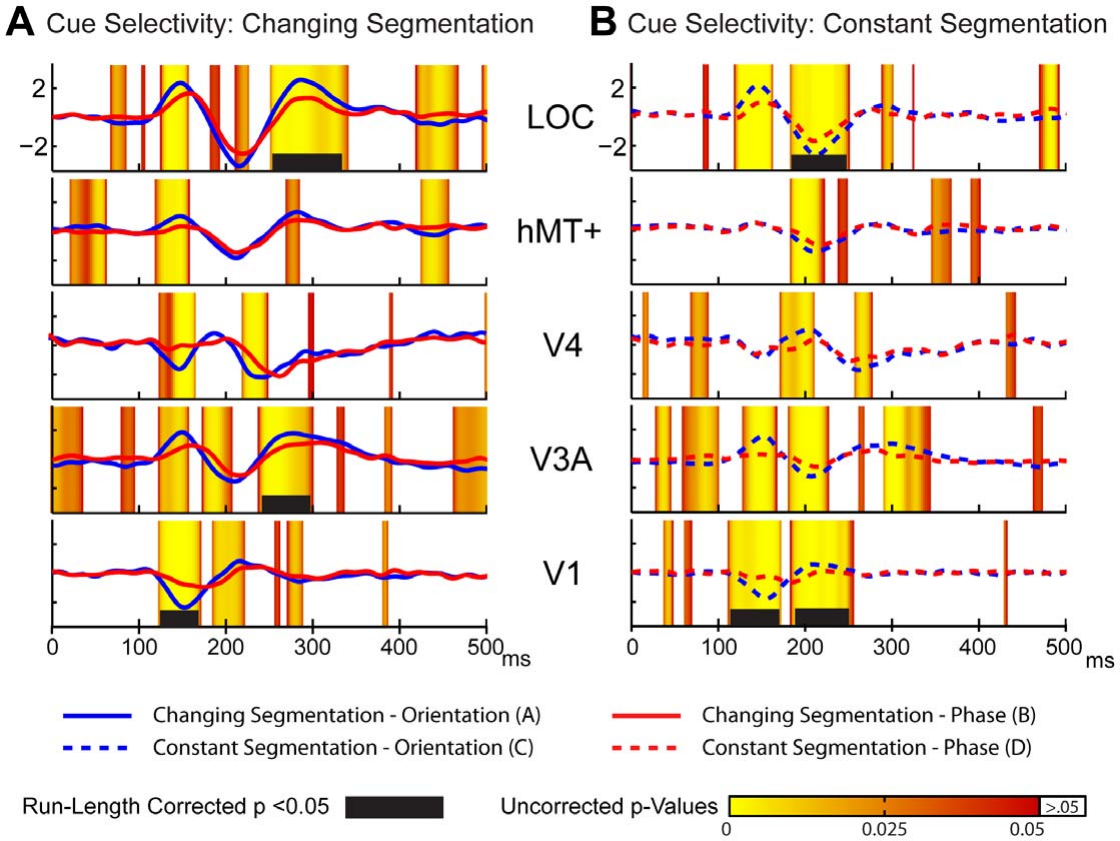
Source-space cue selectivity contrasts. Cue differences for the (**A**) changing and (**B**) constant segmentation conditions in 5 visual area ROIs.

As seen in the sensor-space data, ROI differences to the two cues types occurs earlier than selectivity to the stimulus configuration. The earliest latency at which differential run-corrected activity is present is 114 ms in the V1 ROI for the constantly segmented stimuli (**Figure 7A**), and at 124 ms for the changing segmentation stimuli (**Figure 7B**). By contrast, the earliest significant configural selectivity occurs in V1 at 133 ms. Differences between cues are widespread in the early cortical areas for both the changing and constant segmenting contrasts. For the changing segmentation contrast (solid lines), there are early un-corrected differences present in all the ROIs between ∼125 and 175 ms and later run-corrected differences in V3A and LOC. In comparison to the changing segmentation differences, cue selectivity for constantly segmented textures evokes relatively less early contribution from V4 and hMT+, but is otherwise quite similar.

### Cue-invariance

In our previous work, we showed that several different texture cues led to a similar pattern of cortical activation, indicating that a certain degree of cue-invariance is present in the evoked response from lateral cortex [29]. The overall pattern of activity seen with the present stimuli is similar to our previous findings in that both stimuli produce prominent responses in lateral cortex. To quantitatively compare the responses to the different cues, we computed the difference response between the two tsVEPs (orientation- and phase-defined).

**Figure 8:**
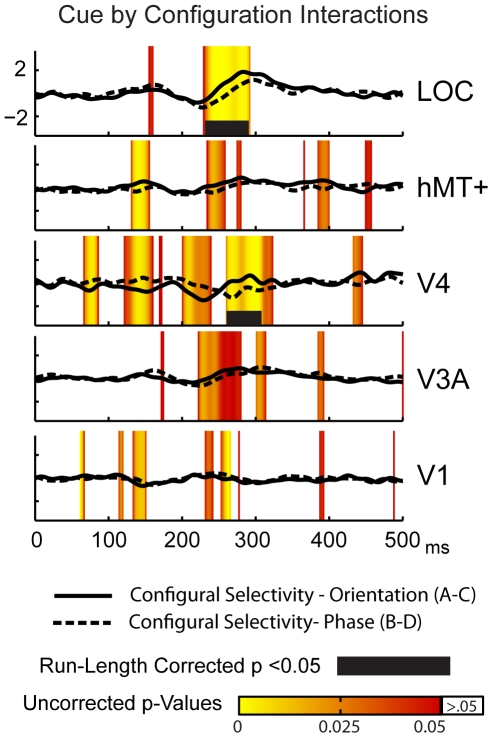
Source-space cue-configuration double difference. Differences between orientation- and phase-defined tsVEPs are restricted to longer latencies (>230 ms) and higher cortical areas (V4 and LOC).

This double difference (**Figure 8**) revealed that while the separate tsVEPs for each type alone produced significant differences across early latencies (e.g. 130–200 ms) there was no differences between these effects at these latencies. Similarly, no differences were seen at any latency in the V1, hMT+ and V3A ROI's. Significant differences were present, however, between 220 and 290 ms in the LOC ROI and between 250 and 300 ms in the V4 ROI. Cue invariant tsVEPs are thus present between 130 and 200 ms and in the V1, V3A and MT+ ROIs, but not at later time-points in the LOC and V4 ROIs.

## Discussion

In the present study we characterize the neural responses to segmenting textured stimuli across the human visual cortex using an fMRI-constrained, EEG source-imaging technique. We observed that segmentation-specific responses were first detected in the V1 ROI for both cue types where small but statistically reliable differences between uniform and segmented stimuli began around 115–130 ms, after an initial period of configuration independent activity. Segmentation-specific activity was then observed in all ROI's except in hMT+. There were subtle differences in the initial pattern of texture-specific responses, with differential activity outside of the V1 ROI being observed more dorsally for the orientation cue and more laterally for the alignment cue. At later time-points both cues produced differential activity in bilateral LOC.

### Response topography, timing and source-distribution: comparison to previous studies

Isolation of tsVEPs has been demonstrated for a host of visual cues including orientation [45], [46], motion [42], stereo cues [47], [48], temporal cues [29], [49], and illusory contours [50]. In general, tsVEPs manifest as a negative polarity potential that reflects the configural selectivity of cortical mechanisms, once responses to low-level features have been subtracted away. In our experiments, evoked responses triggered by texture updates in the central disk region depended strongly on the nature of the background context. In both the changing orientation and phase conditions, responses to the same local contrast transients within the central disk were more negative at the scalp when the context conferred by the background texture led to a change in global segmentation from a uniform field to a segmented one, than when the context signaled a constantly segmented global structure. In our paradigm, the constant segmentation condition serves as a control for local feature responses such as those evoked by a change in orientation of the central disk texture. In the orientation condition, constant segmentation was maintained by the presence of a phase discontinuity when the central region texture was horizontal and by an orientation cue when it was vertical. In the phase condition, segmentation was in the constant segmentation condition was consistently defined on the basis of a phase cue over both updates of the central disk region.

Our stimulation paradigm is similar to that used in the first studies of the texture segmentation VEP [41], [42], and later studies by Caputo et al. [46] and Fahle et al. [48] in that these studies each used a continuous alternation between uniform and segmented global image structure. Our paradigm differs in that our displays have a distinct asymmetry in the perceptual organization of the segmented regions. The older studies used continuous sequences that alternated between uniform fields and texture-defined checkerboards where figure/ground assignment was ambiguous. Our stimuli have an asymmetric figure-ground configuration and an unambiguous perceived depth order — the central disk appears to lie in front of the background. The displays used here generate bilateral response topographies and produce robust configuration-specific responses in the LOC ROI. Previous studies that have used checkerboard configurations [42], [48] have found the tsVEP to be maximal on the occipital midline, rather than over lateral electrodes, as found in the present study. Other tsVEP/MEG studies that have used single figures [4], [26]–[29], [51], [52] have also found lateralized evoked response maxima. When texture segmentation displays with symmetric/ambiguous and asymmetric/unambiguous configurations have been directly compared, both fMRI data [25] and evoked response data [27] suggest that the classic figure-ground configuration of a small figure on a larger background preferentially activates the Lateral Occipital Complex, a region of cortex that is specialized for object processing. Thus the spatial configuration of the stimulus is an important determinant of the network of areas underlying the tsVEP.

The texture-specific response in the earlier tsVEP studies that alternated patterns between uniform and segmented state found a component structure that was dominated by a relative negativity of the segmented state-response at around 200–300 ms [47], [48], [53], [54]. In the present study we also see a dominant relative negativity in the scalp maps in this latency range (see **Figure 3C**). This sensor-level response is maximal bilaterally off the occipital midline and receives contributions from the LOC ROI (see **Figure 6**). In our recordings, the earliest cue-specific activity we record begins on occipital midline electrodes (**Figure 4C**) with a corresponding difference in the V1 ROI beginning between 133 and 143 ms (**Figure 7**). Caputo et al., [46] reported an early tsVEP peak at the occipital midline that approached statistical significance at around 90 ms. Scholte et al., [26], using flashed rather than continuous single stimuli, found their initial texture-specific responses at 90 ms on medial occipital electrodes, consistent with our scalp maps and initial source activity in V1. They attributed this response to boundary detection mechanisms, rather than to mechanisms that represented the surface of the figure. Surface-related activity was first seen on temporal electrodes at around 112 ms. Our stimuli did not distinguish border from surface-related activity, but the sequence of medial to lateral progression is similar, albeit slightly later. The activity we record here in the LOC ROI very likely reflects the surface organization of the stimuli because the simple presence of border discontinuities, without the figure-ground spatial configuration does not robustly activate the LOC [27].

As discussed above, previous studies from our laboratory have used an ROI-based EEG source-imaging approach and frequency tagging technique to study texture segmentation [27]–[30]. Using both orientation and alignment texture cues we identified dorsal and ventral visual areas that were selectively responsive to the figure and background regions, respectively. In addition, across these tasks we observed that the evoked responses attributed to the figure regions were largely similar, independent of the defining cue and the manner in which selective attention was directed. In the present study we expanded these findings to examine the strength, time course and source distribution of the tsVEP generated by orientation and alignment cues. We find only relatively subtle differences in timing and source distribution between the responses to the two cues, Taken together with our previous results from the frequency-tagging method, we suggest that early cortical areas robustly encode both alignment and orientation discontinuities.

#### Mechanisms of texture segmentation

Contextual modulation of the response to the central disk region could arise from either ‘remote’ surround suppression arising from the static background or from local lateral interactions across the border between the disk region and the background. As described below, recent results from cat and macaque V1, along with previous tsVEP results from our laboratory suggest that both processes are active contributors to the tsVEP. The response of V1 cells to stimuli presented within the CRF of the anesthetized cat is largely insensitive to the relative phase of the center and surround, and suppression survives the introduction of a gap between the center and surround [14]. This suggests the presence of a long-range input from the surround. By contrast, in the alert macaque, the arelative phase of the center and surround plays a strong modulatory role, with maximal suppression occurring for perfectly aaligned center-surround configurations that are abutted. Suppression with aligned (collinear) configurations is reduced by the introduction of gaps between the center and surround that are only a small fraction of the CRF size [23], but suppression is nonetheless present. In the anesthetized cat, the gap must cover a much larger area — one that encompasses most of the suppressive surround — before a change in suppression strength is observed [14].

The pattern of evoked responses in the two 90° orientation conditions suggests that the waveform and magnitude of the evoked response to updates of the central region is controlled more by local border discontinuity mechanisms than by a phase-insensitive surround suppression mechanism. If the response was controlled by a purely phase-insensitive surround suppression process, then the responses would not differ in the changing segmentation and constant segmentation conditions because these conditions differ only on the basis of the relative alignment of the surround and center textures in the horizontal state. Responses differ dramatically between these conditions and this suggests that local interactions across the border between the two regions may be dominating our results. In our previous work that used separate temporal frequencies in the center and surround regions of similar texture segmentation displays we were able to show that by introducing gaps between the center and surround region there is a strong nonlinear interaction between texture regions [28]. This interaction is strongest when one state of the display is continuous and is disrupted by gaps that are similar in size to those that Xu and colleagues [23] found could disrupt the effects of a collinear surround on CRF responses. With the single frequency technique used in the present study, this non-linear interaction will project onto the same time-course as the response to the central region and thus we are not able to distinguish the contributions of this nonlinear border process from the local responses arising from the center region itself. The importance of border-region signals in texture segmentation VEP has also been highlighted by others [26], [55].

Taken together, the present results along with data from our previous work and data from cat and monkey suggests the presence of a long-range, phase-independent process that is relatively independent of spatial separation. This process acts in conjunction with an additional phase-dependent (local) component that is restricted to borders that are in close proximity to the CRF [23]. Finally, there is an orientation tuned component of surround suppression that is present even with gaps between the center and surround [13]. It has been suggested that this form of suppression in V1 arises via feedback from higher cortical areas [56] and complements a second, local spatial interaction that occurs across borders. A determination of relationship between this form of interaction and the other types of center-surround interaction just described awaits further study.

In addition, it is worth noting that while numerous texture-segmentation VEP studies have reported an enhancement of late activity for task relevant segmentation stimuli [41], [45], [47], [48], [53], [57], it has also been reported that at least some aspects of the segmentation process proceed automatically without the participant's awareness [1]–[4], [30]. In the present study, we sought to assess time course and circuitry underlying texture-based segmentation under conditions of focused attention. As volitional attention was not manipulated here, the extent and degree to which the underlying visual cortical circuitry activated here is influenced by directed attentional demands, remains an open and important question.

### Cue invariance

As noted above, the borders between objects and surfaces can be defined by a variety of cues and there has been considerable interest in finding cells that can signal border properties, independent of the local information that defines the texture discontinuity. Cue-invariant responses have been seen as early as V1 in macaque [18], [58], [59], (but see [60]). The tsVEP has also been measured across visual dimensions (cues) and varying degrees of cue-invariance have been reported. Bach and Meigen [47] recorded tsVEPs to checkerboards defined by luminance, motion, disparity and orientation. They found that the tsVEP across cue types were much more similar to each other than were the corresponding low-level VEPs that were also recorded. These results were obtained from a single recording channel over the occipital pole. Using multi-channel recordings and a similar range of cue types, Fahle and colleagues [48] also found that tsVEPs were more similar to each other than the corresponding local cue responses, but found more differences in timing and waveform morphology between cues than were reported by Bach and Meigen.

In the present study, we find cue-invariance over the two cue types we studied in the V1, V3A and hMT+ ROIs and between 140 and 220 ms in the V4 and LOC ROIs. The differences in the tsVEP we do measure are small and are confined to long-latencies and higher-order cortex. In our previous study of cue-invariance [29], we did not make direct quantitative comparisons of responses in different cortical areas and we used frequency-domain measures that may have made it difficult to resolve the temporally discrete failures of cue-invariance seen here in **Figure 8**. The differences between our two cues are much smaller than those used in the Bach and Fahle studies, and it would be useful to explore a wider range of stimuli using source-imaging to quantify response profiles in different cortical areas, as it is likely that the degree of cue-invariance may vary over time and across cortical areas.

### Relationship to Figure-Ground segmentation

Differential responses to uniform vs. segmented center-surround configurations could be manifestations of mechanisms that extract the figure-ground relationship, the presence of feature discontinuities, or both. Single-unit recordings in alert behaving macaque have shown that some, but not all, of the early stages of establishing the figure-ground relationship begin in early cortical areas where they are reflected as enhancements of the later, but not earlier portion of the response to the appearance of a figure [61], [62]. Because selectivity for the figure-ground relationship is only seen on the later part of the response, it has been assumed that feedback from higher-level areas helps to confer configuration specificity in early cortex [63]. Support for this conclusion has come from lesion studies showing that ablation of area V4 (but not V2) results in severely impaired in the perception of texture-defined and illusory contours [64]–[67]. Following this line of reasoning [26] have suggested that late signals over occipital pole electrode arise from feedback from higher visual areas. We have some evidence for late responses in the V1 ROI that could arise from feedback because there are responses at earlier latencies in higher-level areas. This pattern of results is only suggestive of feedback however, given that neither we nor [26] have established a functional relationship between responses in different areas and because our late responses in the V1 ROI do not pass our strictest run-length corrected statistical threshold. TMS data [68] have provided a more direct line of evidence for feedback being involved in texture segmentation, but it is not clear whether TMS has disrupted the same signals that are being measured with the tsVEP. It would therefore be useful to combine TMS with EEG source-imaging as a way to trace causal interactions between cortical areas responsible for texture segmentation and figure-ground segmentation.
